# *Bacopa monnieri*: Preclinical and Clinical Evidence of Neuroactive Effects, Safety of Use and the Search for Improved Bioavailability

**DOI:** 10.3390/nu17111939

**Published:** 2025-06-05

**Authors:** Anna Gościniak, Anna Stasiłowicz-Krzemień, Marta Szeląg, Jakub Pawlak, Izabela Skiera, Hanna Kwiatkowska, Natasza Nowak, Krzysztof Bernady, Piotr Trzaskoma, Oskar Zimak-Krótkopad, Judyta Cielecka-Piontek

**Affiliations:** 1 Department of Pharmacognosy and Biomaterials, Poznan University of Medical Sciences, Rokietnicka 3, 60-806 Poznan, Poland; agosciniak@ump.edu.pl (A.G.); astasilowicz@ump.edu.pl (A.S.-K.); 2 The Student Scientific Society of Poznan University of Medical Sciences, Department of Pharmacognosy and Biomaterials, Poznan University of Medical Sciences, Rokietnicka 3, 60-806 Poznan, Poland; m.szelag21@wp.pl (M.S.); jpawlak2910@wp.pl (J.P.); izabela.skiera@wp.pl (I.S.); kwiatkowska.han@gmail.com (H.K.); 85251@student.ump.edu.pl (N.N.); 85334@student.ump.edu.pl (K.B.); piotrtrzaskoma242@gmail.com (P.T.); 3 Department of Pharmacology and Phytochemistry, Institute of Natural Fibres and Medicinal Plants, Wojska Polskiego 71b, 60-630 Poznan, Poland; oskar.zimak-krotkopad@iwnirz.pl

**Keywords:** *Bacopa monnieri*, memory enhancement, Alzheimer’s disease, neuroprotection

## Abstract

*Bacopa monnieri*, also known as Brahmi or Waterhyssop, is a plant used in Ayurveda for its memory-enhancing properties and control of blood sugar levels. It contains active compounds such as alkaloids, saponins, and cucurbitacins, which have various biological activities. The plant has been studied for its potential in treating Alzheimer’s disease, Parkinson’s disease, attention deficit hyperactivity disorder (ADHD), and depression. Animal studies have shown promising results in reducing symptoms and protecting against neurodegeneration. Concerning safety, *Bacopa monnieri* has been found to be generally non-toxic, with no serious side effects reported. However, interactions with certain medications and contraindications in conditions like hyperthyroidism should be considered. Further research is needed to determine optimal dosages and ensure safety, especially for pregnant and breastfeeding women.

## 1. Introduction

**Figure 1:**
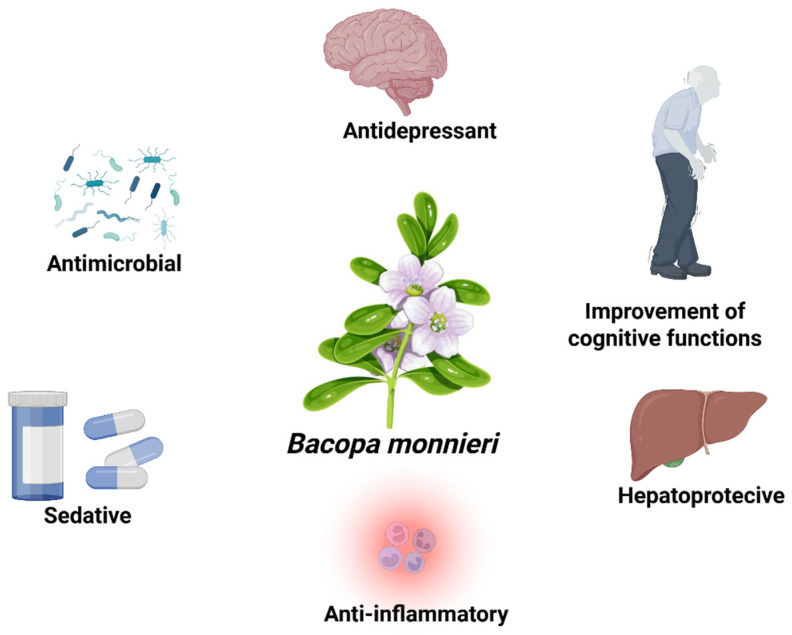
Schematic representation of the main neuropharmacological effects of *Bacopa monnieri*.

*Bacopa monnieri* is a plant that has been used for 5000 years in Ayurveda, the traditional Indian medicine. It belongs to the *Scrophulariaceae* family and grows naturally in the southeastern regions of Asia (India, Australia, Sri Lanka). This plant, characterized by its obovate leaves and small white flowers, is used in traditional medicine to enhance memory. Additionally, it is used to control blood sugar levels. In the book *Charaka Samhita*, which describes Ayurvedic culture, Bacopa is presented as a plant for treating various mental disorders and is referred to as “medhya rasayana”, which translates to “rejuvenating (or revitalizing) herbs” that are believed to enhance memory, mental health, and intellect, as well as to promote long life and rejuvenation [1,2]. Therefore, in Ayurveda, Bacopa was used for insomnia, epilepsy and as an anti-anxiety agent. However, this herb has broader applications in various diseases due to the presence of compounds such as alkaloids and saponins, for example, bacosides A, which may be used to support the treatment of gastric ulcers or Alzheimer’s disease (Figure 1) [3,4].

Despite human studies showing that *Bacopa monnieri* positively affects learning and word recall, the FDA (Food and Drug Administration) has not approved the medical use of this herb and additionally issued a warning in 2019 to dietary supplement manufacturers producing *Bacopa monnieri* preparations not to make any therapeutic claims regarding this herb [5].

*Bacopa monnieri* has traditionally been used to enhance memory, and this study aims to review the potential use of the plant in the treatment of Alzheimer’s disease, Parkinson’s disease, ADHD, and depression. While some modest improvements in memory performance and benefits in conditions like Alzheimer’s and depression were observed, the results were inconsistent across tests, indicating the need for more robust, standardized clinical studies [3]. Studies on the activity of plant raw materials are particularly challenging due to the various types of extracts, which differ in composition and concentration of active compounds. The complexity of plant matrices and the diversity of extraction methods further complicate the standardization and reproducibility of results. A review of current research and discussion of safety aspects will help guide future studies in this area.

## 2. Active Compounds

*Bacopa monnieri* is rich in secondary metabolites, including a number of alkaloids (herpestine, brahmine), saponins (bacosides, betulic acid, and hersaponin), alcohols, flavonoids, sterol glycosides, sugars, amino acids and cucurbitacins [4,6].

### 2.1. Saponins

**Figure 2:**
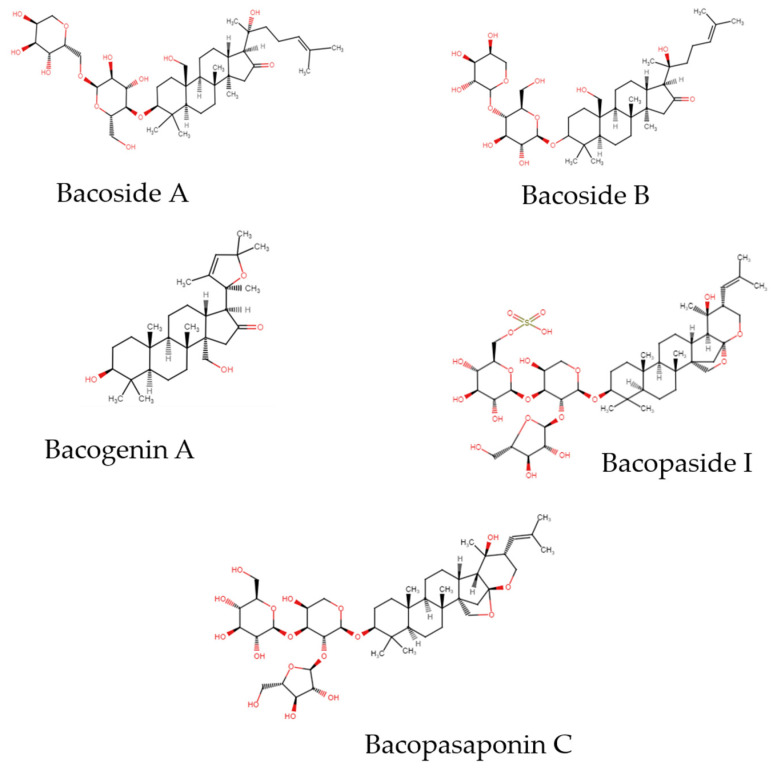
Chemical structures of the major active compounds found in *Bacopa monnieri*.

The major chemical compounds from *Bacopa monnieri* are dammarane-type triterpenoid saponins with jujubogenin or pseudojujubogenin moieties as aglycones. These compounds can be found throughout the plant, in the leaves, and in stems [5]. Saponins in the plant include bacosides (Figure 2), Bacopasides, or betulinic acid, which play essential roles in neuronal health and are collectively present at a concentration of up to 6% of the dry weight of the plant [7]. Among these, bacoside A stands out as a major saponin fraction, comprising approximately 38% of the dry mass of standardized methanolic extract [8], and includes four saponin glycosides: Bacopaside II, Bacopaside X, Bacoside A3 and Bacopasaponin C. Bacoside A demonstrated anti-tumor activity in in vitro studies on cell lines as well as in vivo studies conducted on mice [9]. Moreover, Bacoside B (which contains Bacopaside IV, V, N1 and N2 and varies in optical rotation with bacoside A) can be isolated and is considered one of the bioactive marker compounds for this species. A yield of 0.65% (dry weight basis) has been reported for isolated bacoside B [10], which is considered a bioactive marker compound for this species. Bacogenins A1–A5 are the acid-hydrolyzed derivatives of bacosides. The major component is bacogenin A4—ebelin lactone. Bacopasides I-XII interact with sterols and are involved in membrane disruption. Among bacopasaponins, Bacopasaponin C comprises 0.3–0.6% of the ethanolic extracts of *Bacopa monnieri*. Bacopasaponin C is a glycoside of pseudojujubogenin with glucose and rhamnose as sugar units, and it shows anti-dandruff properties. The HPLC-based study quantified individual components of bacoside A in *Bacopa monnieri*. Bacoside A3 ranged from 0.14% to 0.85%, and Bacopaside II from 0.12% to 0.69%. These values reflect regional variation and confirm measurable quantities of bacoside A components in the plant [4,11,12].

### 2.2. Alkaloids

The primary alkaloids found in *Bacopa monnieri* include brahmine, which is believed to contribute to its neuroprotective effects. Brahmine was one of the first alkaloids to be isolated from the plant. Herpestine is another notable alkaloid found in *Bacopa monnieri*. It contains nitrogen atoms and has a complex molecular structure typical of alkaloids. Herpestine may influence neurotransmitter systems, such as serotonin and dopamine, which are crucial for mood regulation and cognitive functions. These constituents have been identified in ethanolic extracts and contribute to the plant’s pharmacological profile. Herpestine is of interest for its potential neuromodulatory effects, while monnierin is associated with cognitive-enhancing activity. Their detection underscores the chemical complexity of *Bacopa monnieri* and supports its traditional use in cognitive and neuroprotective therapies [5].

### 2.3. Cucurbitacins

**Figure 3:**
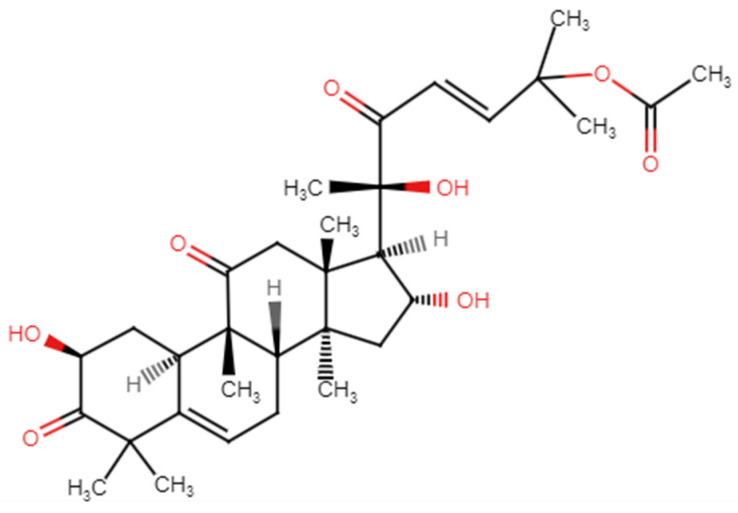
Structure of cucurbitacin B present in *Bacopa monnieri*.

Cucurbitacins are a class of highly oxygenated tetracyclic triterpenoids known for their bitter taste and pharmacological properties. Cucurbitacins have a tetracyclic triterpenoid core structure consisting of four fused rings derived from cucurbitane. Bacopa contains cucurbitacins such as cucurbitacin B and cucurbitacin E, which contribute to its biological properties. The structure of cucurbitacin B is presented in Figure 3. Cucurbitacin E has recently been reported to possess inhibitory effects on the growth of human colon, breast, lung, and central nervous system cancer cell lines [13,14]. Cucurbitacin B induces cell cycle arrest and inhibits angiogenesis and tumor cell migration, indicating its potential as a promising candidate for anticancer drug development. It is important to note that cucurbitacin B is not a primary bioactive constituent of *Bacopa monnieri*, whose pharmacological effects are mainly attributed to bacosides [11,15].

## 3. Biological Activity

**Figure 4:**
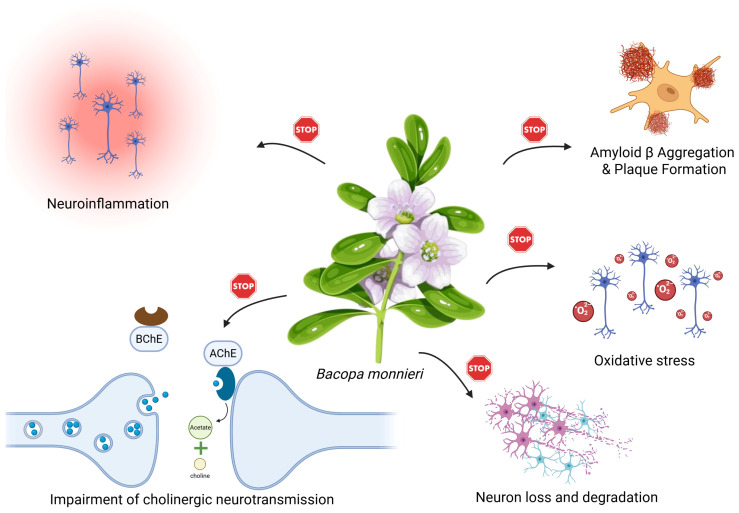
Schematic representation of the neuroprotective mechanisms of *Bacopa monnieri*.

*Bacopa monnieri* shows great potential for multi-targeted activity, primarily due to its rich profile of bioactive compounds. It is particularly renowned for its impact on the nervous system, as it exhibits neuroprotective effects on multiple levels of neural functioning and across various neurodegenerative pathways. The neuroprotective actions of *Bacopa monnieri* are mediated through a diverse range of mechanisms (Figure 4).

Foremost among them is its potent antioxidant activity, which mitigates oxidative stress by neutralizing reactive oxygen species and inhibiting lipid peroxidation, thereby preserving neuronal integrity [16,17]. The plant also displays anti-amyloidogenic properties, reducing the aggregation and deposition of amyloid-beta peptides—an essential mechanism in the context of Alzheimer’s disease [18]. Additionally, Bacopa acts as an acetylcholinesterase inhibitor [19], enhancing cholinergic transmission through increased acetylcholine levels, which contributes to improved memory and cognitive performance. It further influences neuroinflammation and mitochondrial function, reducing neuronal apoptosis and promoting cellular resilience [20]. The above, along with additional mechanisms, are discussed in detail in the following subsections.

### 3.1. Alzheimer’s Disease

Alzheimer’s disease (AD) is a chronic, progressive neurodegenerative disease characterized by irreversible damage to the central nervous system. It leads to cerebral dementia, associated with the loss of many cognitive functions such as memory, speech, and the ability to learn new skills. The source of this disease is not entirely clear, which makes it challenging to find appropriate medications to prevent its rapid progression. Currently, cholinesterase inhibitors and glutamate antagonists are used. In the course of the disease, abnormal deposition of beta-amyloid protein in the brain is observed in the form of plaques and Tau protein. They induce the development of free radicals that impair glucose transport in nerve cells, leading to damage and death of neurons [21]. Death of neurons causes dysfunction of the cholinergic system—deficits in cortical choline acetyltransferase concentrations, reduced choline release, and reduced acetylcholine secretion [22,23].

Treatment of Alzheimer’s disease relies on delaying the progression of the disease by potentiating transmission in the cholinergic system. There is no effective causal treatment. Alzheimer’s disease is a neurodegenerative disease that affects the elderly and is estimated to account for 60% of all dementia cases in people over the age of 65. The statistics do not indicate improvement, as it is estimated that by 2050, the number of patients may increase two- or threefold. However, the troublesome side effects on the gastrointestinal tract, which lead to damage to the stomach lining and peptic ulcers, often do not allow for long-term pharmacological therapy in Alzheimer’s disease [6].

In an in silico study conducted by Roy et al. [24], it was shown that out of 17 compounds found in *Bacopa monnieri*, 2 saponins, Bacopasaponin G and Bacopasaponin N, exhibited inhibitory effects on Caspase-3 and tau-protein kinase, which are factors related to Alzheimer’s disease. These findings suggest potential therapeutic activity; however, further in vitro and in vivo studies are necessary to confirm this.

Using methanol extracts has shown the anti-amyloidogenic potential of Brahmi by significantly affecting the dissociation of amyloid protein aggregates [25]. A study on cultured rat cells confirmed these findings, showing a reduction in beta-amyloid deposition in the brain. The neuroprotective effect of *Bacopa monnieri* was likely due to its ability to inhibit acetylcholinesterase activity rather than mitigating glutamate-induced toxicity. Additionally, neurons treated with the extract exhibited lower levels of reactive oxygen species, suggesting a reduction in intracellular oxidative stress and an extension of neuronal lifespan. The extract also demonstrated antioxidant properties and inhibited lipid peroxidation [26].

Following the experiment on 72 mice, it was shown that supplementation with *Bacopa monnieri* (100 mg/kg for 180 days) significantly improved cognitive function. In healthy mice, memory, concentration, and learning ability were enhanced, while in Alzheimer’s mice, cognitive performance was restored to near-normal levels. These effects were confirmed by the Morris water maze test, with statistical analysis indicating significant differences between groups (F significant, *p* < 0.01) [27].

In a randomized, double-blind clinical trial, 60 healthy, elderly volunteers taking 300 mg and 600 mg of Brahmi showed reduced acetylcholinesterase activity, which resulted in improved attention and memory. Hence, *Bacopa monnieri* may be useful in the treatment of Alzheimer’s disease as well as attention deficit disorder. Moreover, no side effects or toxicity were observed [28].

**Figure 5:**
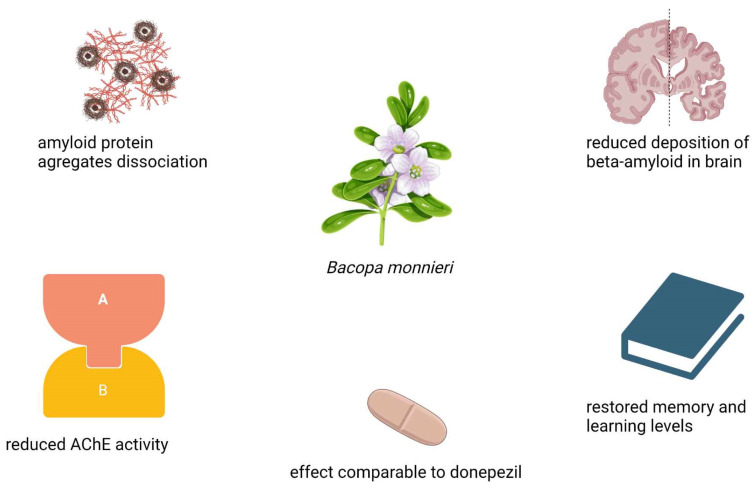
Schematic representation of the effects of *Bacopa monnieri* in patients with Alzheimer’s disease.

While *Bacopa monnieri* has demonstrated promising cognitive benefits in clinical studies, its efficacy in Alzheimer’s disease (Figure 5) must be evaluated in the context of existing pharmacological treatments. Current drugs for Alzheimer’s, such as acetylcholinesterase inhibitors (AChEIs), primarily aim to mitigate cognitive decline by enhancing cholinergic transmission or modulating excitotoxicity. In a 52-week, randomized, double-blind study, Brahmi demonstrated an effect comparable to donepezil, a drug used to treat Alzheimer’s disease. The efficacy and safety of *Bacopa monnieri* (300 mg) and synthetic donepezil 10 mg were compared in 48 patients with Alzheimer’s disease and mild cognitive impairment. They showed no significant difference between them after 1 year of treatment [29].

### 3.2. Parkinson’s Disease

Parkinson’s disease is a neurodegenerative disorder that consists of selective death of nigral dopaminergic neurons and the presence of Lewy bodies containing ɑ-synuclein. These factors lead to motor symptoms such as tremors and muscle stiffness as well as cognitive impairment. The disease is caused by a combination of numerous factors, including age, genetic liability, brain injuries, and exposure to toxins [30].

**Figure 6:**
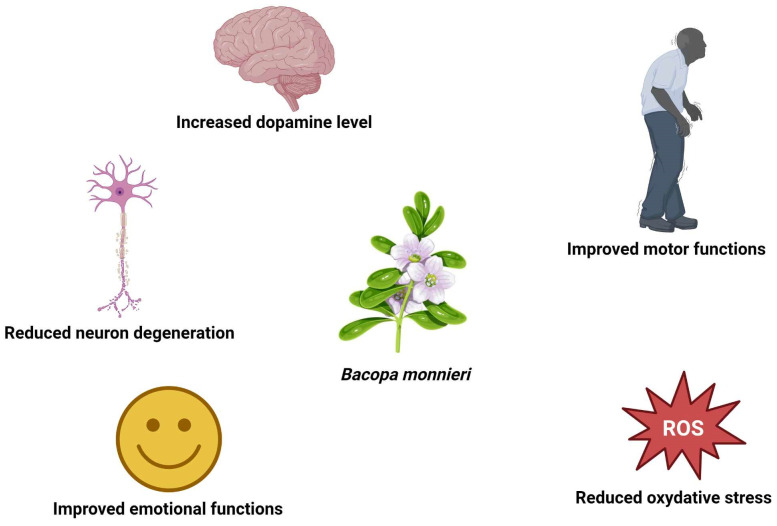
Schematic representation of the effects of *Bacopa monnieri* effects in Parkinson’s disease.

*Bacopa monnieri* has shown promising neuroprotective properties in the context of Parkinson’s disease (Figure 6). In silico studies using molecular docking confirmed the potential ability of Bacoside-A to interact with the active site of Parkinson’s disease protein 7 (DJ-1) [31] and Bacopaside-XII to inhibit Kelch-like ECH-associated protein 1 (KEAP1) [32]. DJ-1 and KEAP1 play a role in the accumulation of Lewy bodies by causing oxidative stress, and its inhibition may result in improvement of the symptoms.

Experiments conducted on invertebrates also provided promising results. Transgenic *Caenorhabditis elegans* expressing ɑ-synuclein and *C. elegans* exposed to 25 mM 6-hydroxydopamine were administered a *B. monnieri* tincture (100 g DW/650 mL 96% ethanol) diluted ten times in *E. coli* OP50 liquid culture for 48 h. Transgenic *C. elegans* treated with *Bacopa monnieri* developed significantly reduced accumulation of ɑ-synuclein and lipoperoxidation compared to the control group. Furthermore, *Bacopa monnieri* reduced degeneration of dopaminergic neurons in the group exposed to 6-hydroxydopamine [33].

In other study, transgenic Parkinson’s *Drosophila melanogaster* was exposed to 0.25, 0.5, and 1.0 µL/mL *Bacopa monnieri* extract for 24 days. It was observed that *Bacopa monnieri* treatment reduced oxidative stress and delayed motor impairment compared to the untreated control group [34].

In a study on male albino mice conducted by Singh et al. [32], animals received oral administration of *Bacopa monnieri* extract (40 mg/kg) for 30 days. They were injected intraperitoneally with MPTP (1-methyl-4-phenyl-1,2,3,6-tetrahydropyridine) (15 mg/kg) from days 15 to 30 to induce Parkinson’s-like neurodegeneration. Mice treated with *Bacopa monnieri* showed improved motor performance and reduced dopaminergic neuron degeneration compared to the MPTP-only group. Brain analysis revealed lower oxidative stress markers and pro-apoptotic proteins, along with increased antioxidant activity and Bcl-2 expression.

Similar results were observed in mice treated with 40 mg/kg *Bacopa monnieri* extract for 3 weeks followed by intraperitoneal injections of 30 mg/kg MPTP from the second to third week and in the group simultaneously exposed to MPTP and *Bacopa monnieri*, which indicates both the neuroprotective and neurorescue potential of *Bacopa monnieri* [32]. An experiment conducted by Shinomol et al. [35] on rats exposed orally to 180 mg/kg *Bacopa monnieri* extract for 80 days and intraperitoneally to 2.5 mg/kg rotenone from the 20th to 80th day also resulted in increased noradrenaline, adrenaline, dopamine, and serotonin and reduced MAO levels in brain tissue.

The double-blind, placebo-controlled, parallel trial conducted by Fuentes dos Santos et al. [36] on 20 volunteers with Parkinson’s disease suggests that *Bacopa monnieri* could be useful in PD therapy. Patients were administered 225 or 400 mg of *Bacopa monnieri* extract a day for 90 days. A significant improvement in both motor and emotional function was observed, which resulted in increased quality of life. However, to confirm the potential of *Bacopa monnieri*, more clinical trials are needed.

### 3.3. Attention Deficit Hyperactivity Disorder (ADHD)

Attention deficit hyperactivity disorder (ADHD) is one of the most common neurodevelopmental disorders in children, manifesting itself in problems with inattention, hyperactivity, and impulsivity, which result in learning and social difficulties [37].

Dave et al. [38] conducted a study on 27 children who met the criteria for ADHD. Participants took 225 mg of *Bacopa monnieri* extract a day for 6 months. The symptoms of ADHD, excluding social problems, were significantly reduced after treatment, especially self-control, which increased in 89% of the children. Additionally, levels of restlessness in 85% of participants were reduced. Results clearly confirm that *Bacopa monnieri* has the potential for application in ADHD therapy. That improvement may be caused by the influence on the dopamine level, which decreased in the prefrontal cortex in patients diagnosed with ADHD.

### 3.4. Depression

Major depressive disorder (depression) is a common and significant mental disorder that impacts one’s emotions, thoughts, and behaviors in multiple ways. It affects up to 21% of the population. The disorder is characterized by feeling dismal, displaying a lack of enthusiasm for pursuits that were previously engaged in with eagerness, a significant change in sleeping schedule (sleeping too much or too little), and thoughts of death and/or suicide. The symptoms are not identical in every patient, and it requires at least 2 weeks of observation to be clinically diagnosed [39]. Seeking natural remedies might play a vital role since they have fewer side effects [40].

Researchers aimed to evaluate the antidepressant effects of the methanolic extract of *Bacopa monnieri* through various behavioral models designed to demonstrate antidepressant activity, with tests yielding positive results. Swiss albino mice were subjected to various behavioral models, including the forced swimming test, the locomotor activity test, and the tail suspension test [41]. The forced swimming test assessed depressive behavior in mice given either water, methanolic extract, or imipramine [42]. During the test session, the animals were exposed to stressful conditions for 5 min. The study revealed that the methanolic extract of *Bacopa monnieri* significantly decreased the immobility duration in the forced swimming test (*p* < 0.001) [41]. The Open Field Maze test measured locomotion, rearing, and defecation. Mice treated with extract or imipramine showed significant increases in all parameters compared to controls (*p* < 0.001) [43].

A study on Charles Foster rats used a learned helplessness test with shock pretreatment and avoidance training. Rats received *Bacopa monnieri* extract (20 or 40 mg/kg) or imipramine (15 mg/kg). The control group received a carboxymethyl cellulose suspension. Bacopa significantly reduced escape failures and increased avoidance responses after 5 days [8].

### 3.5. Anxiety

**Figure 7:**
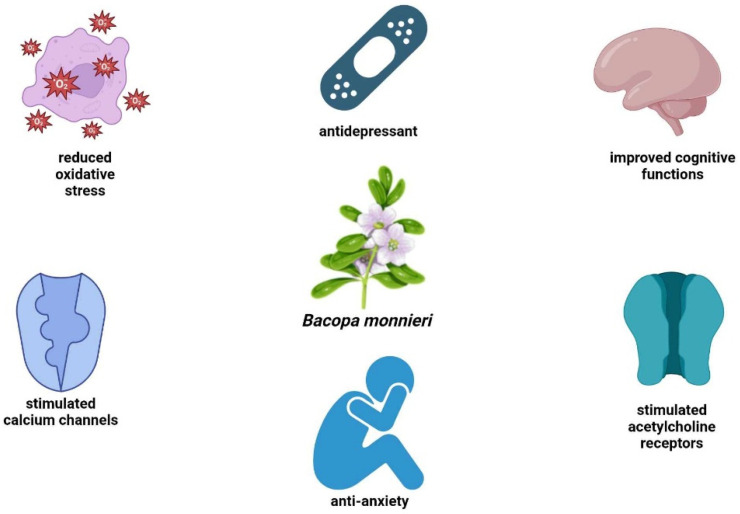
Schematic representation of the anxiolytic effects of *Bacopa monnieri*.

Anxiety is a common reaction to stress, but anxiety disorders go beyond ordinary feelings of nervousness or uneasiness. They are characterized by excessive fear or apprehension. They represent the most prevalent category of mental disorders, impacting nearly 30% of adults during their lifetime. Simple anxiety may go beyond to more severe disorders such as panic disorder [44]. *Bacopa monnieri* has demonstrated multiple beneficial effects in individuals suffering from anxiety (Figure 7). A dose of 150 mg of a standardized extract of *Bacopa monnieri* (Bacognize) administered twice daily for six weeks was tested in a randomized group of people subjected to neuropsychological tests [45]. The results indicated a significant improvement in the Bacopa group compared to the placebo-treated group. Considerable differences were shown in the logical memory test and the reduction in distractibility. The improvement could be a result of the antioxidant effect and action on both calcium channels and acetylcholine, which are Bacopa-dependent.

*Bacopa monnieri* provides a wide range of potential therapeutic effects, as summarized in Table 1, where the collected studies reflect its promising activity demonstrated across different study types and disease models.

### 3.6. Other Bioactive Properties

*Bacopa monnieri*, in addition to its neuroprotective properties, exhibits a range of other health-promoting effects. It is well-known for its adaptogenic properties, helping the body cope with stress and maintain physiological balance [4]. The plant demonstrates anti-inflammatory effects, which may assist in reducing inflammation within the body, as well as antioxidant properties that protect cells from oxidative stress [3]. Furthermore, studies suggest its beneficial impact on the cardiovascular system, including lowering blood pressure and supporting vascular health. *Bacopa monnieri* also exhibits hepatoprotective properties, aiding in liver regeneration and shielding it from toxins. Additionally, some research highlights its potential anticancer effects and its ability to boost immune system function. This wide spectrum of properties makes *Bacopa monnieri* a compelling subject for further investigation into its systemic health benefits [9].

### 3.7. Limitations of Studies

Despite the promising results obtained in numerous studies on *Bacopa monnieri*, there are a number of important limitations that need to be taken into account when interpreting their results and potential clinical application. Most of the available studies are in silico analyses, in vitro experiments, or animal model studies. Although they point to potential mechanisms of action and beneficial effects of *Bacopa monnieri* on neuroprotective parameters, a systematic assessment of the quality of this evidence is lacking. In many cases, these studies are characterized by small samples, lack of blinding, and short durations, limiting the generalizability of the results to the human population. Translating the results of preclinical studies into clinical effects remains a major challenge. In silico and in vivo studies indicate anti-amyloidogenic and antioxidant effects, among others, but clinical studies confirming these mechanisms in Alzheimer’s patients are lacking. Even well-designed human clinical trials, e.g., comparing Brahmi with donepezil, are few and need to be replicated in wider study groups [29]. One key limitation is the considerable variability in the composition of *Bacopa monnieri* extracts. Differences due to the extraction method (ethanolic vs. methanolic), the source of the plant material, the culture conditions, or the type of standardized active compounds used (e.g., bacosides) affect the reproducibility of the results and their comparability. Non-standardized preparations make it difficult to replicate test results and may have varying pharmacological and safety profiles.

## 4. Safety of Use

Evaluating the safety of plant raw materials is important for many reasons. It allows assessing the impact on both human health and the environment. *Bacopa monnieri*, which is widely known in Ayuverdhist medicine, has also been of scientific interest for its effects on the nervous system. Currently, however, there is still a lack of research on the safe use of the raw materials in pregnant and breastfeeding women, which is important from the point of view of fetal and child development in the early months of life.

### 4.1. Doses Used

Studies to date do not report strict daily doses to ensure adequate safety, as *Bacopa monnieri* is generally a non-toxic plant. Ethanol extract was most commonly used in the studies. Doses are usually in the range of 300 to 600 mg daily. Based on a clinical study conducted, among others, by Benso et al., doses of 320 mg and 640 mg were shown to have a positive effect on human cognitive function (memorization, eye tracking, etc.). Slightly stronger effects were observed in those who received the 640 mg dose [46]. In a study conducted by Dave et al. [38], a safe dose for children aged 6 to 12 years was developed. The study involved 31 children with ADHD. The children were given a dose of 225 mg per day for 6 months. The dosage has proven not only safe but also effective in eliminating ADHD symptoms in children within a certain age range. Clinical studies have demonstrated that daily doses of 300 to 600 mg of *Bacopa monnieri* extract standardized for the amount of bacosides, equivalent to 5 to 10 g of the dried herb, can enhance cognitive function and alleviate symptoms of anxiety and depression [47]. These effects are typically observed after 12 weeks of consistent supplementation. However, further research is needed to refine these dosage recommendations and to assess the long-term safety and efficacy of Bacopa monnieri across diverse populations.

### 4.2. Toxicity In Vitro

The purpose of the toxicity study is to provide the necessary information on the safety of the herbal product before evaluating its potential benefits in clinical trials. Sireeratawong et al. conducted a study to assess the chronic and acute toxicity of *Bacopa monnieri* extract in rats. In the acute toxicity study, female rats were divided into two groups. Group I (control) was administered distilled water at 1 mL/kg, and Group II (test) was administered *Bacopa monnieri* extract at 5000 mg/kg. The rats were subjected to close observation for 14 days. Body weight and hematological, biochemical, and histopathological parameters were evaluated. As a result, no significant changes were observed in the aforementioned parameters. In addition, the high dose of 5000 mg/kg did not contribute to the death of rats. Although the control and treatment groups’ body weights differed somewhat at the start of the experiment, this difference was not statistically significant. Body weight changed little but significantly only after 14 days. Since it can be the result of variations in food consumption, this little increase in body weight in the treated animals might still be regarded as normal. Rats treated with *B. monnieri* showed no significant histological changes in any of their internal organs, including the kidneys and liver. Doses of 30, 60, 300, and 1500 mg/kg/day of *Bacopa monnieri* extract were used for chronic toxicity testing. The control group was given distilled water, and the test group was given appropriate doses of *Bacopa monnieri* extract for 270 days. In the conducted study, there were no significant changes which would indicate toxicity of the extract [48]. The clinical study, which was conducted on 23 volunteers, showed no alarming changes indicating toxicity of the preparation containing *Bacopa monnieri*. Each participant received a 300 mg tablet and a 450 mg tablet for 30 days. Each dose was consumed for 15 days. Studies that were conducted before and after the use of the product showed no serious side effects. In some cases, gastrointestinal disorders in the form of diarrhea have appeared. Based on studies to date, there are no contraindications to the withdrawal of preparations containing *Bacopa monnieri* extract resulting from the toxicity of the plant material [49].

### 4.3. Possible Side Effects

At the moment, there are no reports of serious adverse effects. The most common side effects are gastrointestinal disorders. A study by Morgan and Stevens evaluating the effects of *Bacopa monnieri* on the memory of the elderly found that nausea, excessive abdominal cramps, and frequent bowel movements appeared after prolonged use [50].

Singh and Singh conducted a study to assess the effect of *Bacopa monnieri* extract on mouse fecundity. *Bacopa monnieri* was found to have the ability to induce reversible suppression of spermatogenesis and fertility in mice. However, it does not affect libido or cause toxic effects [51].

Administration of a dose of 500 mg/kg to rats resulted in decreased appetite and increased concentrations of albumin, urea, nitrogen, and aspartate aminotransferase, among others. Nevertheless, no notable changes in organ weights were observed at the end of the 90-day study [52].

### 4.4. Contraindications

Despite the high tolerance and safety of using *Bacopa monnieri* extract, there are also contraindications to using preparations containing this herb. Studies have shown that due to the inhibition of cytochrome isoenzymes (CYP2C9, CYP2C19, CYP1A2, CYP2D6, CYP3A4), interactions occur between the drug and the herb. Such interaction occurs between the extract of *Bacopa monnieri* and amitriptyline (a drug with antidepressant and sedative effects). *Bacopa monnieri*, by inhibiting cytochrome isoenzymes CYP3A and CYP2C responsible for the metabolism of amitriptyline, reduces the first-pass metabolism of this drug and additionally decreases the clearance of the mentioned drug after oral administration. Inhibition of cytochrome isoenzymes by Bacopa may lead to side effects of drugs metabolized by the inhibited isoenzymes. It has been shown that due to the herb–drug interaction, side effects occurred with the simultaneous intake of Bacopa extract and agomelatine—an antidepressant (metabolized by CYP1A2); back pain and excessive sweating were observed. A similar interaction is shown with the combination of *Bacopa monnieri* and Moclobemide—an antidepressant (metabolized by CYP2C19); myocardial infarction was observed as a side effect [53].

Particular caution should be exercised in cases of hyperthyroidism, as an in vivo study on male mice demonstrated that *Bacopa monnieri* leaf extract (200 mg/kg) significantly affects T4 (thyroxine) levels. The concentration of this hormone increased by 41%, which is undesirable in hyperthyroidism, where this hormone is already elevated. Attention should be paid to medications affecting T4 levels when simultaneously using *Bacopa monnieri* to avoid further increasing thyroxine levels [54].

Another study demonstrated that gastrointestinal side effects from using *Bacopa monnieri* extract are caused by the cholinergic action of this plant, leading to cholinergic stimulation of the gastrointestinal system, resulting in effects such as increased tension and peristalsis of the stomach and intestines, abdominal cramps, increased bowel movements, and peptic ulcer disease. On the other hand, *Bacopa monnieri* may also be useful in peptic ulcer disease not only due to its potential stress-relieving effects, which can mitigate the exacerbation of the disease, but also through its influence on the growth of *Helicobacter pylori*. Research indicates that chronic stress can weaken the gastric mucosa, making it more susceptible to *H. pylori* infection, a key factor in the development of peptic ulcers [55]. *Bacopa monnieri* ethanol extract showed antiulcer activity in a mouse model at a dose of 400 mg/kg body weight. At this dose, therapeutic effectiveness comparable to omeprazole, used in the treatment of gastric ulcers, at a dose of 20 mg/kg body weight was observed. The result is associated with increased mucin secretion and reduced desquamation of mucosal cells [56].

The cholinergic action of *Bacopa monnieri* results in increased acetylcholine levels (acetylcholinesterase inhibition), so special attention should be paid when simultaneously administering *Bacopa monnieri* extract and acetylcholinesterase inhibitors (AChE-I) currently used in treating Alzheimer’s disease, to avoid a radical increase in acetylcholine levels. Due to its function, high acetylcholine levels may lead to bradycardia, so individuals predisposed to slow heart rates should consult their doctor about whether *Bacopa monnieri* extract supplementation is safe [50]. Early studies suggest that the concomitant use of *Bacopa monnieri* and galantamine may have a synergistic effect on cognitive function. Still, there is a need for further research into the potential risk of side effects and pharmacodynamic interactions [57]. Considering also its acetylcholine-enhancing properties, it can cause stomach ulcers, worsening of asthma, and chronic obstructive pulmonary disease or urogenital tract obstruction, which indicates monitoring of patients at risk [50]. It is also important to monitor patients taking medications for hypothyroidism through the effect of Bacopa on the release of thyroid hormones to avoid excessive concentrations of the drug in the body, which can cause health risks for patients [54].

### 4.5. Bacopa Extract in At-Risk and Sensitive Groups

The use of Bacopa extract among vulnerable groups such as children, the elderly, or pregnant women is of particular interest because of their physiological differences. Despite its promising properties for supporting the nervous system, information on its safety of use in the previously mentioned populations is limited.

Among the clinical trials using Bacopa extract in recent years, two studies by the research group Kean et al. conducted on children aged 6–14 years can be found. The study tested the effectiveness of the extract in children diagnosed with ADHD. During the study, only a few cases of gastrointestinal discomfort were reported, which were short-lived and did not result in treatment discontinuation [58,59]. Other studies include two conducted on children aged 4–18 years and 6–12 years. The studies focused on the extract’s properties for increasing children’s intellectual abilities and reducing symptoms of ADHD. During the study, as in the previously cited ones, several cases of gastrointestinal disturbances were found, including vomiting and upset stomach lasting about three days, after which no further side effects were observed [60,61].

Another vulnerable population is the elderly. A recent study conducted by Best et al. on middle-aged and elderly patients (35–65 years) used a mixture containing *Bacopa monnieri*. Despite the sensitivity of this group to a number of medicinal substances, in this case, no side effects or intolerance to the received preparation were found during the study or during the follow-up phase [62]. Some studies reported the appearance of gastrointestinal disorders, in particular nausea, increased bowel movements and abdominal cramps, or, less frequently, dry mouth. Other side effects included fatigue and “flu-like” symptoms [50,57,63,64].

In the case of the last sensitive population, which is pregnant and lactating women, there is a lack of clinical studies on the safety of *Bacopa monnieri* extract. Only animal studies are available that do not address the aspect of safety during pregnancy and lactation [65,66]. As a result, many researchers note that women should not use Bacopa-containing supplements during this time. Further clinical studies evaluating the potential benefits and risks in this population are needed [67].

## 5. Bioavailability

Bacoside A is one of the main active compounds found in *Bacopa monnieri*. However, its water insolubility results in low bioavailability when administered orally. Additionally, it has a bitter taste, which is unacceptable for patients when using oral preparations. Research has been conducted to increase the bioavailability of bacoside A and simultaneously remove its bitter taste for more effective action of *Bacopa monnieri* and dose reduction. An inclusion complex with cyclodextrin (β-CD) was utilized to address both drawbacks of bacoside A. Bacoside A/β-CD complexes were created in various molar concentrations (1:1, 1:2, 1:3, 1:4, 1:5) using the co-precipitation method—each sample was tested separately. It was shown that the best solubility was exhibited by the sample with a *Bacopa monnieri/*β-CD ratio of 1:4 (solubility increased threefold). Additionally, the bitter taste of Bacoside A was masked [3].

In vivo studies conducted on mice attempted to enhance the anti-amnesic effect of the *Bacopa monnieri* extract using a new formulation, namely with the use of phospholipids. The study involved mice suffering from amnesia due to the natural aging process. A complex was created using Bacopa extract and L α-phosphatidylcholine to increase absorption in intestinal fluids. Due to their biocompatibility with phytochemical compounds, phospholipids caused the *Bacopa monnieri* preparation in complex form to demonstrate greater anti-amnesic efficacy than the ordinary preparation of this plant at the same dose (40 mg/kg, p.o.). In the Morris water maze test, a significant reduction in escape latency time (ELT), as well as an increase in time spent in the target quadrant (TSTQ), was observed in the phospholipid complex group, indicating improved acquisition and recall of spatial memory. Additionally, the complex caused a much stronger inhibition of AChE activity in the brain compared to the conventional *Bacopa monnieri* extract, suggesting a more effective enhancement of cholinergic transmission, which is crucial for memory processes [68].

The phospholipid complex contained high concentrations of active bacosides (Bacoside-A: 51.92%; Bacopaside-I: 43.2%; Bacopaside-II: 48.6%), which maintained effective concentrations of active compounds for a longer period compared to the conventional extract, indicating formulation stability and efficacy, and suggesting a prolonged half-life (t ½) and slower elimination. Following oral administration, the phospholipid complex showed higher serum concentrations of bacosides (Bacopaside I/BPC complex at 12.21 μg/mL vs. BE at 10.41 μg/mL; Bacopaside II/BPC complex at 12.28 μg/mL vs. BE at 10.38 μg/mL). This represents a 17–18% increase in maximum concentration (Cmax) for the bacosides. The physical intercalation of bacosides into the phospholipid layers enhances their solubility and facilitates transport across lipid-rich biological barriers such as the intestinal membrane and the blood–brain barrier. The presence of L-α-phosphatidylcholine improves molecular compatibility and absorption of phytocompounds in the gastrointestinal tract [4].

## 6. Modern Methods of Administration

Modern drug delivery methods are becoming increasingly popular in the context of phytotherapy. *Bacopa monnieri*, thanks to its pleiotropic effect, can be administered to patients in a variety of ways, increasing both its effectiveness and bioavailability. Researchers are interested not only in oral administration but also in transdermal or injectable forms. A major role is played by nanotechnology, which has made it possible to develop nanoparticles that make it possible, among other things, to overcome the blood–brain barrier. In addition, scientific reports also inform about other methods of administration of active ingredients such as bilosomes, ethosomes, and polymerosomes. With these innovations, *Bacopa monnieri* is gaining prominence in the context of a holistic approach to health and well-being.

### 6.1. Bilosomes

*Bacopa monnieri* extracts must cross the blood–brain barrier in order to work in the central nervous system. For this purpose, an attempt was made to place the extract in nanovesicles, forming liposomes and bilosomes. Two methods were used to obtain these formulations: solvent evaporation and thin-film hydration with phosphatidylcholine. Bilosomes and liposomes were analyzed in detail and characterized by homogeneous particle size and appropriate zeta potential. Bacopa extract at a dose of 200 mg/kg was used in a study on Swiss mice with memory loss. The use of bilosomes resulted in more effective memory improvement compared to liposomes. This is due to better bioavailability and greater stability in simulated gastric and intestinal juice conditions. It is worth noting that 1/10th of the dose considered in the acute toxicity evaluation was used for the study. This is due to the vastly improved bioavailability, which still requires more advanced studies such as radio-labeling [69].

### 6.2. Ethosomes

Pertaining to skin aging, Brahmi plays a crucial role in maintaining a youthful and healthy appearance due to its antioxidant properties, which protect the skin from free radicals—one of the main contributors to premature aging. Brahmi also contains compounds that stimulate collagen production and improve skin elasticity, reducing the appearance of wrinkles. This effect is attributed to the plant’s chemical composition, particularly the presence of bacosides and alkaloids. Studies have been conducted in which *Bacopa monnieri* extract was incorporated into nanolipoidal vesicles, such as ethosomes, to enhance its penetration into the deeper layers of goat skin, aiming to increase collagen production and reduce elastin degradation in the dermal layer. Ethosomes exhibit flexibility and deformability, allowing them to penetrate the disordered stratum corneum and reach deeper skin layers. Through the use of ethosomes, *Bacopa monnieri* extract was able to penetrate up to a depth of 30–40 µm in goat skin. Permeation analysis demonstrated that systems following zero-order kinetics provide continuous substance release over an extended period, enhancing therapeutic effects. Therefore, ethosomes are suitable for transdermal delivery of herbal extracts [70].

### 6.3. Nanoparticles

Bacoside II, a bioactive compound present in *Bacopa monnieri*, exhibits in vitro cytotoxic effects against various cancer cell lines. It has been shown to induce cell cycle arrest and apoptosis in colorectal cancer cell lines while also inhibiting the migration and tubulogenesis of endothelial cell lines, thereby demonstrating anti-angiogenic properties. Research investigating the effects of PLGA-PEG-encapsulated Bacopaside II nanoparticles (BM1NPs), characterized by prolonged retention time in systemic circulation, on C6 glioma cells have revealed that BM1NP accelerates particle internalization within these cells. This suggests its potential anti-tumor activity through increased ROS levels, inhibition of cell proliferation, and induction of apoptosis in C6 glioma cells [71].

Studies were also conducted in which, similarly to the aforementioned approach, formulated solid lipid nanoparticles (SLNs) were used to facilitate the transport of the bacoside-rich extract across the blood–brain barrier. Swiss albino male mice were utilized in these experiments. The results demonstrated that SLNs exhibited greater efficacy in alleviating neurodegeneration compared to the extract alone, indicating their potential as a delivery system for bacosides across the blood–brain barrier in the treatment of Alzheimer’s disease [72].

Similar experiments have been conducted in the context of epilepsy treatment, where the extract was converted into nanoparticles (BM3NP and BM4NP). Following the administration of BM3NP and BM4NP, KA-treated rats exhibited a significant reduction in epileptic spikes, a decrease in sleep latency, and an increase in NREM sleep duration, demonstrating the potential of nanoparticles in neutralizing epileptic seizures [73]. Further clinical studies may provide additional confirmation of the efficacy of herbal extracts in this regard.

### 6.4. Limitations of Modern Delivery Systems

Although advanced delivery systems show promise in improving the effectiveness of *Bacopa monnieri*, they also have some important limitations. One major issue is the lack of direct comparisons with traditional forms such as oral extracts or tinctures. Many studies report positive results using modern systems, but because they use different methods, doses, and models, it is hard to say which method works best overall. More consistent studies using the same standards are needed to compare their effectiveness. Another challenge is related to regulations and safety. Products that use nanotechnology must go through detailed safety testing, especially because their long-term effects on the body are not yet fully understood [74]. Also, the process of obtaining approval for these products can be complex and varies between countries, making it harder to bring them to market [75]. Advanced drug delivery systems, such as nanoparticles, liposomes, and other nanocarriers, often entail higher production costs due to their complexity and the specialized technologies required for their manufacture. These increased costs can pose significant barriers to large-scale production and widespread adoption [76]. Advanced drug delivery systems can be problematic if they do not take familiar forms that are acceptable to patients. The route and form of administration are key factors influencing patient acceptability. Acceptability is significantly affected by characteristics such as appearance, ease of swallowing, taste (palatability), and handling of the dosage form, particularly in the case of solid oral formulations. Attributes like size, shape, and texture also play an important role in shaping patient preferences, which can impact treatment adherence and overall effectiveness [77]. New or unfamiliar technologies, as well as alternative routes of administration, may cause discomfort, uncertainty, or resistance among users. In summary, while modern delivery systems offer exciting possibilities, more research is needed to compare them fairly, ensure their safety, and make them practical and acceptable for everyday use.

## 7. Legal Aspects

In the European Union (EU), *Bacopa monnieri* is considered a pharmacopeial plant, and a monograph by the European Medicines Agency (EMA) is currently being developed for this plant. *Bacopa monnieri* can be legally used as an ingredient in dietary supplements; however, it is not classified as a novel food since it was used in dietary supplements in the EU before 15 May 1997, and is therefore not subject to Regulation (EU) 2015/2283. However, applications other than dietary supplements—such as *Bacopa monnieri* in food—may require authorization under the Novel Food Regulation (EU) 2015/2283 before being placed on the EU market. Additionally, general food safety regulations must be considered, including Regulation (EC) No 178/2002, which establishes the general principles and requirements of food law, as well as other regulations that may restrict or regulate the use of the product in specific EU member states [78]. The U.S. Food and Drug Administration (FDA) has not issued specific regulations regarding *Bacopa monnieri* dietary supplements and has not approved this plant as a drug. Therefore, it has not made any official statements regarding its safety or effectiveness. The FDA has issued several general regulations concerning dietary supplements, such as the Dietary Supplement Health and Education Act (DSHEA) and the Current Good Manufacturing Practice (CGMP). DSHEA defines dietary supplements and establishes rules for their labeling and sale, while CGMP sets quality standards for the manufacturing process of dietary supplements. The FDA only intervenes if a supplement is found to be unsafe or is marketed illegally. Therefore, it is recommended that a healthcare professional be consulted before taking any dietary supplement containing *Bacopa monnieri* [79]. Preparations containing *Bacopa monnieri* are regulated in Canada as Natural Health Products (NHPs). They are, therefore, subject to the relevant regulations established by Health Canada, such as the Natural Health Products Regulations and the Natural Health Products Ingredients Database. *Bacopa monnieri* is used in dietary supplements; however, there are restrictions on the maximum dosage, which depend on factors such as age and health status. Additionally, manufacturers and importers must obtain the appropriate license to conduct such activities and comply with quality manufacturing standards [80,81].

## 8. Conclusions and Perspectives

*Bacopa monnieri* shows a wide range of neuroprotective effects, including antioxidant, anti-inflammatory, and cholinergic actions, which may help support cognitive and emotional functions. Results from preclinical studies and early clinical trials suggest its potential as a complementary treatment for neurodegenerative and neurodevelopmental disorders. To move forward, research should focus on connecting laboratory findings with real clinical benefits. While comparisons with standard drugs like donepezil are encouraging, they need to be backed by well-designed clinical trials that directly compare treatments and explore how *Bacopa monnieri* works in the human body. Modern delivery methods such as bilosomes and nanoparticles offer promising ways to improve the effectiveness of *Bacopa monnieri* by enhancing its absorption and stability. However, these approaches still need to be carefully tested for safety, patient acceptance, and scalability for wider use. Future studies should aim to confirm how *Bacopa monnieri* works in the human brain, identify who benefits most from its use, and ensure that extracts are consistent in quality and composition. Building a stronger link between lab research and clinical practice will be key to unlocking the full therapeutic potential of this plant.

## Figures and Tables

**Table 1:** Summary of study results on various diseases.

Disease	Type of Study	Study Group	Duration of the Study	Dose	Conclusions	Reference
Alzheimer’s disease	In silico	-	-	-	Inhibitory effects on the CASP-3 and TPK I	Roy et al. [3]
	In silico	-	-	-	The anti-amyloidogenic effect from dissociation of amyloid protein aggregates	Subramanian et al. [4]
	In vitro	Cortical cell cultures were prepared from 18-day-old rat fetuses	72 h	10–100 μg/μL (extract: 18 kg DW/triple percolation with 95% ethanol, 8 h)	The anti-amyloidogenic effect, limiting deposition beta-amyloid in brain	Limpeanchob et al. [6]
	In vivo	72 healthy and AD mice	180 day	100 mg/kg (methanolic extract)	Memory and learning deficits restored to normal levels;	Kunte and Kuna [27]
	Randomized, double-blind clinical trial	60 healthy volunteers	12 weeks	300 mg, 600 mg (methanolic extract)	memory enhancement and reduction in AChE activity	Peth-Nui et al. [28]
	Randomized, double-blind clinical trial	48 patients with AD and mild cognitive impairment	45 months	300 mg (standardized extract)	Effect comparable to donepezil	Prabhakar et al. [29]
Parkinson’s disease	In silico	-	-	-	Interaction of Basocide-A with active site of DJ-1	Chandrasekar et al. [21]
	In silico	-	-	-	Inhibition of KEAP1 by Bacopaside-XII	Singh et al. [32]
	In vivo	*Caenorhabditis elegans*	48 h	*B. monnieri* tincture (100 g DW/650 mL 96% ethanol), diluted ten times in E. coli OP50 liquid culture	Reduction in α-synuclein accumulation, degeneration of dopaminergic neurons, and lipoperoxidation	Jadiya et al. [22]
	In vivo	Transgenic PD *Drosophila melanogaster*	24 days	0.25; 0.5; 1.0 µL/mL (extract: 30 g DW/300 mL acetone)	Delayed motor impairment, reduction in oxidative stress, and brain apoptosis	Siddique et al. [24]
	In vivo	24 male albino mice	30 days	40 mg/kg (extract: 500 g DW/1000 mL ethanol)	Better results in motor tests, reduced lipid peroxidation, conjugated dienes, caspase-3, Bax protein, dopaminergic neuron degeneration, increased Bcl-2 protein, antioxidant activity, nitrate level in nigrostriatal system	Singh et al. [25]
	In vivo	32 Swiss albino mice	3 weeks	40 mg/kg (commercial extract)	Better results in motor tests, reduced lipid peroxidation, increased GSH, dopamine, and nitrate level in the nigrostriatal system	Singh et al. [23]
	In vivo	24 male Wistar rats	80 days	180 mg/kg (ethanolic extract)	Increased noradrenaline, adrenaline, dopamine, serotonin, reduced MAO in brain tissue	Shinomol et al. [26]
	Double-blind placebo-controlled, parallel trial	20 volunteers with Parkinson’s disease	90 days	225; 400 mg a day (commercial extract)	Improvement in quality of life, emotional and motor functions	Fuentes dos santos et al. [36]
ADHD	Open-label trial	27 children meeting the criteria for ADHD	6 months	225 mg a day (commercial extract)	Significant reduction in ADHD symptoms except for social problems	Dave et al. [38]
Anxiety	Double-blind placebo-controlled, parallel trial	60 students aged 19–22 having basic computer literacy	6 weeks	150 mg two times a day (standardized extract, 15.57 of bacopasaponins (%*w*/*w*)	Significant effect on cognitive functions	Kumar et al. [45]
Depression		Inbred Charles Foster albino rats	5 days	20 and 40 mg/kg, once a day (methanolic extract)	Decreased escape failures and increased avoidance responses	Sairam et al. [8]
	In vivo	Swiss albino mice	5 min	50, 100 and 200 mg/kg (sample extract) 30 mg/kg (standard drug)	Confirmed antidepressant-like effect of methanolic extract of *B. monniera*	Mannan et al. [41]

## Data Availability

Not applicable.

## Abbreviations

The following abbreviations are used in this manuscript:

ADHD	Attention deficit hyperactivity disorder
FDA	Food and Drug Administration
AChE-I	Acetylcholinesterase inhibitors
T4	Thyroxine
KEAP1	Kelch-like ECH-associated protein 1
MPTP	1-methyl-4-phenyl-1,2,3,6-tetrahydropyridine
DJ-1	Parkinson disease protein 7
DSHEA	Dietary Supplement Health and Education Act
CGMP	Current Good Manufacturing Practice
